# Suction-assisted Tissue Acquisition by Cholangioscopy: A Novel Technique to Improve Diagnosis of Indeterminate Biliary Strictures

**DOI:** 10.1055/a-2955-9845

**Published:** 2026-09-22

**Authors:** Tomazo Antonio Prince Franzini, Marcelo Klotz Dall'Agnol, André Orsini Ardengh, Miriam Chinzon, Luiza Bicudo de Oliveira, Pedro Henrique Veras Ayres da Silva, Danielle Azevedo Chacon, Fauze Maluf-Filho

**Affiliations:** 1 Department of Gastroenterology 117265 Hospital das Clinicas da Faculdade de Medicina da Universidade de São PauloSão PauloSPBrazil; 2 Department of Pathology 117265 Hospital das Clínicas da Faculdade de Medicina da Universidade de São PauloSão PauloSPBrazil

## 

**Video 1** Suction-assited biopsies during cholangioscopy.


Indeterminate biliary strictures remain challenging to diagnose. Although digital single-operator peroral cholangioscopy (DSOC) enables targeted biopsy under direct visualization, tissue acquisition may be suboptimal. 1 2 3 We describe a simple suction-assisted cholangioscopy-guided biopsy technique.

A 52-year-old woman presented with jaundice and weight loss. An outside CT report described a hilar mass causing biliary obstruction. As the images were unavailable for review, EUS with fine-needle aspiration (FNA) and ERCP were performed on the same day. EUS showed a lesion at the hepatic duct confluence extending into the proximal common bile duct (CBD), with intrahepatic ductal dilation ( Fig. 1a,b ). ERCP confirmed a proximal CBD stricture, and biliary stents were placed ( Fig. 2 ). FNA was nondiagnostic because of scant necrotic material. DSOC was then performed because of suspected biliary extension and revealed villous projections with irregular vessels in the CBD, highly suspicious for malignancy ( Fig. 3 ).

**Fig. 1:**
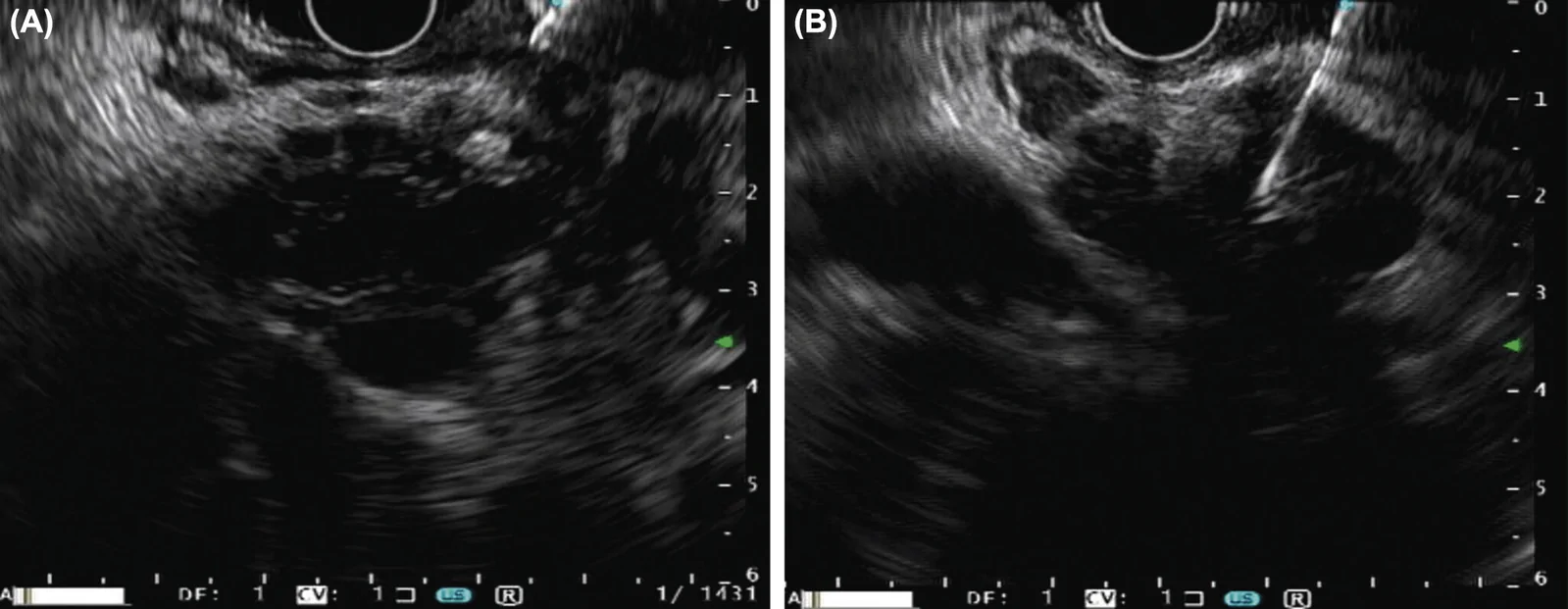
( **A** ) Endoscopic ultrasound (EUS) demonstrating a 70 × 30-mm heterogeneous hypoechoic lesion at the hepatic duct confluence. ( **B** ) EUS-guided fine-needle aspiration of the lesion.

**Fig. 2:**
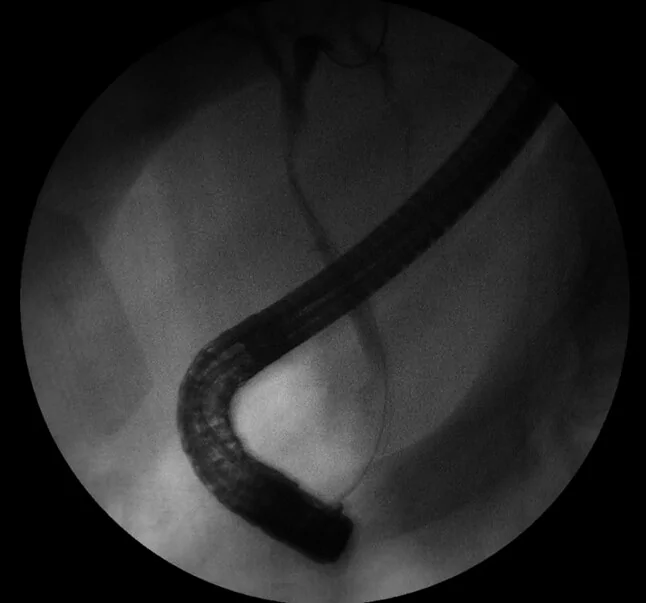
ERCP showing a proximal common bile duct stricture below the hepatic duct confluence.

**Fig. 3:**
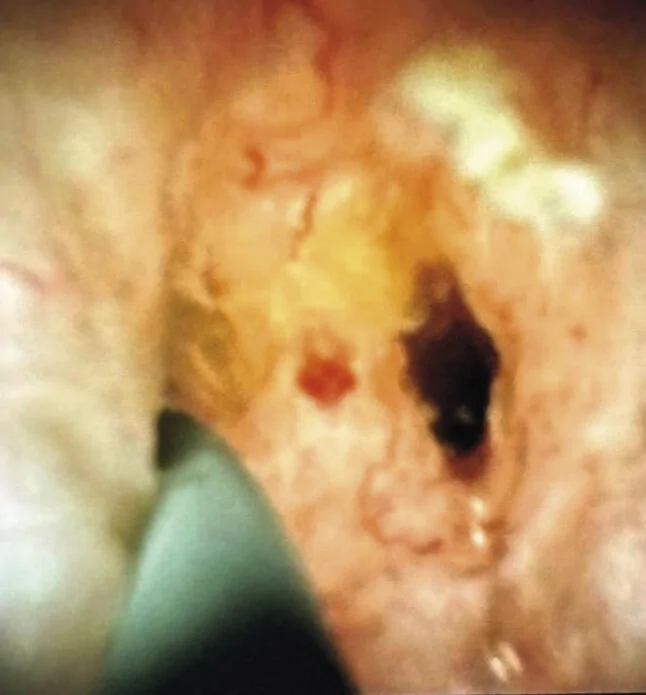
Digital single-operator cholangioscopy revealing villous projections with irregular vessels in the common bile duct.

After cholangioscopic assessment, five suction-assisted tissue acquisition by cholangioscopy (SAT-C) and five standard biopsies were obtained from suspicious areas in the right intrahepatic duct and CBD. Standard biopsies were obtained under direct visualization. For SAT-C, the forceps were positioned over the target area, and saline was continuously aspirated with a 20-mL syringe through the irrigation channel after closing the Tuohy-Borst valve. This induced ductal collapse, allowing the forceps to grasp the mucosa more effectively ( Video 1 ). All specimens were reviewed by an expert pathologist.

SAT-C yielded larger specimens and diagnosed cholangiocarcinoma in malignant-appearing areas, whereas standard biopsies showed only inflammatory changes. Both techniques showed chronic inflammation in the right intrahepatic duct. No procedure-related adverse events occurred.

SAT-C is a simple, low-cost technique that may improve tissue acquisition in indeterminate biliary strictures. Prospective studies are needed to assess diagnostic performance and reproducibility.
